# Association between internet use and social isolation among nursing home residents: a chain mediation model

**DOI:** 10.1186/s12877-025-06656-4

**Published:** 2025-11-11

**Authors:** Qiyuan Huang, Huangqin Liu, Wan Cheng, Sheng Wu, Song Ge, Yuanfeng Lu, Jinyi Wu, Bingqian Ou, Huimin Xiao

**Affiliations:** 1 School of Nursing, Fujian Medical University, No. 1 Xuefu North Road, University Town, Fuzhou, Fujian China; 2 School of Informatics, Xiamen University, Xiamen, China; 3 Department of Natural Sciences/Nursing, University of Houston-Downtown, Houston, Texas USA

**Keywords:** Social isolation, Internet use, Social network, Social support, Social well-being, Nursing home, Path analysis

## Abstract

**Background:**

Social isolation poses a significant threat to the health of older adults. However, there has been a lack of attention to the relationship between Internet use and social isolation. This study aimed to clarify the specific impact paths among Internet use, social network, social support, social well-being, and social isolation in nursing home residents.

**Methods:**

This was a cross-sectional study. A total of 441 nursing home residents in Fujian and Shandong Provinces, China were surveyed between February 2023 and January 2024 using convenience sampling. Guided by the convoy model, a hypothetical theoretical model was developed and tested in this sample. Structured questionnaires were used to collect data on demographics characteristics, Internet use, social isolation, social network, social support, and social well-being. Multiple stepwise linear regression was employed to identify factors influencing social isolation. Additionally, path analysis was conducted to examine the chain mediating effects of social networks, social support, and social well-being on the relationship between Internet use and social isolation.

**Results:**

The total score of social isolation of the participants was 13.47 ± 3.58. Internet use, social isolation, social network, social support, and social well-being were all positively correlated. The results of multiple stepwise linear regression analysis showed that marital status, number of children, pre-retirement occupation, health, social network, social support, and social well-being were the main influencing factors of the social isolation of nursing home residents. Mediation effect analysis showed that Internet use has a non-significant direct effect on social isolation. The indirect effect of Internet use on social isolation was significantly exerted through social network, social support, social well-being, and the chain mediating pathways among them.

**Conclusion:**

Encouraging nursing home residents to use the Internet is beneficial for their social connections and well-being. It can improve older adults’ social networks, social support, social well-being, and alleviate social isolation.

**Supplementary Information:**

The online version contains supplementary material available at 10.1186/s12877-025-06656-4.

## Background

Social isolation is defined as a state that individuals lack a sense of social belonging and engagement with others, which is an important indicator of social relations among older adults [1]. Globally, about a quarter of older adults are currently suffering from social isolation [2]. In China, it has been reported that nearly one-third of nursing home residents experience social isolation [3]. Social isolation has posed a significant threat to health in term of higher mortality rates [4], chronic diseases [5], declines in mental and cognitive health [6], and excessive healthcare resource consumption [7]. Therefore, the World Health Organization has identified addressing the social isolation of older adults as one of the key themes for action during the United Nations Decade of Healthy Ageing (2021–2030) [8].

The Internet has been integrated into various aspects of senior life, profoundly changing the ways of production and living. Digital media has expanded older adults’ social connections, facilitating their integration into social activities, thereby enhancing their social well-being and alleviating social isolation [9]. Robust evidence exists regarding associations among Internet use, social relationships, and social well-being. First, Internet use can have an impact on social isolation. More frequent Internet use can reduce social isolation among older adults [10]. This may be because the Internet can facilitate more connections between older adults and family members, provide a strong sense of belonging, and thus reduce social isolation [11]. However, some studies have drawn that frequent Internet use may lead older adults to develop digital addiction, which exacerbates social isolation [12, 13]. Therefore, more evidence is needed to clarify the association between Internet use and social isolation. Second, the Internet can transcend temporal and spatial constraints, offsetting the shrinkage of older adults’ social networks caused by declining physical function, thereby enhancing the potential for social support. For example, Heo et al. noted that the Internet provides a digital extension for older adults’ daily communication and social interactions [14]. Close social connections can be maintained through remote interactions via mobile devices. However, some researchers are concerned about the negative impact of Internet use on older adults’ social connections. For instance, since Internet use can satisfy various needs, older adults’ offline social activities may consequently be reduced [15, 16]. Third, the experience of Internet use may also affect the social well-being of older adults. Social well-being refers to an individual’s sense of happiness and satisfaction in society. It reflects positive aspects of human well-being through interaction with others and the community at large [17]. Lee et al. argued that Internet use helps strengthen emotional bonds and enhances well-being [18]. The way Internet use enhances social well-being depends on older adults’ choices regarding the functions of the Internet. Lifshitz et al. looked into the connection between different Internet activities and well-being in later life. They found that only online leisure activities can predict well-being by enhancing life satisfaction and reducing depression levels [19]. In view of this, not all online activities have a positive impact on social relationships and well-being. The impact of Internet use on social isolation remains inconclusive. Hence, it is necessary to fully explore the internal mechanisms through which older adults’ Internet use affects their social networks, social support, social well-being, and social isolation.

**Fig. 1:**
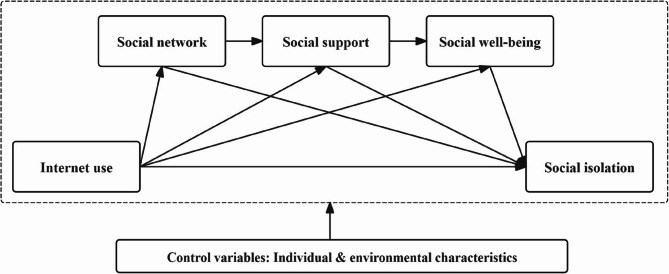
The hypothetical theoretical model

The convoy model provides a theoretical framework for understanding the influence of individuals’ social relationships on social well-being and social isolation [20]. It explains social isolation as a poor health outcome resulting from a combination of narrow social network scope, weak social support, and reduced social well-being. The shrinking of social networks and diminished social support may contribute to a decline in objective social connections among older adults. Meanwhile, lack of sufficient social connections could exacerbate the decrease in individuals’ social well-being [21]. It is the combined effect of these subjective and objective factors that determines the onset of social isolation. Previous study has used the model to explore the mechanism by which social connection affects well-being and health outcomes [22]. However, the Internet as a key social medium for modern human interaction. Few studies have employed the convoy model to investigate the mechanisms by which older adults’ Internet use affects their social relationships, social well-being, and social isolation. Therefore, building on the convoy model and a literature review [23], a preliminary theoretical model was formulated to explore the factors contributing to social isolation among nursing home residents, as well as the relationship between Internet use and social isolation (Fig. 1). The possible transmission mechanisms by which Internet use affects social isolation among older adults may be explained from two perspectives. On the one hand, Internet use improves the mental health, well-being, and emotional support of older adults. Positive subjective perceptions can reduce social isolation. On the other hand, Internet use improves social networks and social support for older adults. Good social relationships also decrease social isolation. Individual and environmental characteristics (e.g., socio-demographic and residential information) will be used as control variables to enhance the robustness of the model. Using this theoretical model, we can provide a more in-depth assessment of the factors that determine and influence social isolation. This study intended to investigate the relationships and mechanisms among the five components of Internet use, social network, social support, social well-being, and social isolation, based on the convoy model. Most importantly, this study sought to increase public awareness of Internet use and social isolation among nursing home residents and provide evidence-based support for innovative social isolation digital intervention research in the future.

## Methods

### Study design

This was a descriptive, cross-sectional study that used chained mediation modeling to clarify the association between Internet use and social isolation among nursing home residents. With this approach, we can identify the direct, indirect, and total effects between the variables. The Strengthening the Reporting of Observational Studies in Epidemiology (STROBE) checklist (Appendix file 1) was followed for this study.

### Participants and setting

Convenience sampling was used to recruit nursing homes from two private, two public, and one public-private partnership settings in Fujian and Shandong Provinces, China, for this study. All nursing homes have passed service quality evaluations of mandatory national standards for aged-care institutions. Participants were recruited through poster postings and public presentations in the study settings. The inclusion criteria for the investigated participants were as follows: (i) aged ≥ 60; (ii) stayed in nursing home ≥ 30 days; (iii) able to communicate in Mandarin Chinese. The exclusion criteria were as follows: (i) cognitive impairment (Short Portable Mental Status Questionnaire ≥ 3); (ii) taking anti-anxiety or anti-depressant medication; (iii) severe visual or hearing impairment.

For the sample size of the path analysis, we conducted a priori power analysis to determine the required number of participants. The equation of the sample size was given by Equation (i) [24]. In this study, significance level (*α*) = 0.05, significance test statistic (*µ*) = 1.96 according to the statistics table. Referring to the reference index [23], population proportion ($\mathrm\pi$) = 0.38, permissible error (*δ*) = 0.05. The minimum sample size of this study was 362. Overall, 441 participants’ data were analyzed.

### Instruments and measures

#### Participant characteristics

A structured questionnaire was developed to obtain participants’: (a) Demographic characteristics (e.g. age, sex, educational level); (b) Economic characteristic was reflected by socioeconomic status (SES), which was measured through a systematic cluster analysis of “educational level”, “monthly fee” and “occupation before retirement” [25]. Based on the clustering combinations of inter-sample distances (*d* = 10) in the dendrogram generated by IBM SPSS 26.0, we divided SES into the lower, middle, and upper strata; (c) Health characteristics, including any chronic diseases and self-assessed health status (responses ranging from “1” very poor to “5” very well); (d) Residential characteristics (e.g. admission preparation, residential form); and (e) Technology characteristics, including technical devices, Internet access, perceived technical usefulness, and perceived ease of use. The measurement of perceived technical usefulness and perceived ease of use referred to the 8-item questionnaire with two dimensions proposed by Venkatesh [26]. It used a seven-point Likert scale ranging from 1 (completely disagree) to 7 (completely agree). Responses across the 4 items were summed to obtain the total score, with higher scores indicating higher degrees of perceived technical usefulness or perceived ease of use.

#### Social isolation

The Social Isolation Scale (SIS) in older adults was developed by Nicholson in 2019 [27]. This study used the Chinese version of the SIS translated by Hui P [28]. The Chinese version of the SIS consists of 6 items and 2 domains, connectedness and belongingness. It uses a five-point Likert scale, with a total possible score of 0–24. A lower score indicates a higher risk of social isolation. In this study, the Cronbach’s α was 0.813, which is considered acceptable.

#### Social network

Social network was measured by using the Chinese version of the Lubben Social Network Scale (LSNS-6-C) [29]. The scale includes six items which measures the size of active and intimate networks in kinship ties and nonkin ties. Using a five-point Likert scale, with a total possible score of 6–30. The higher the score, the larger the size of the individual’s social network. The Cronbach’s α coefficient for the LSNS-6-C in this study was 0.902.

#### Social support

Social support was assessed using the Perceived Social Support Scale [30]. This 12-item questionnaire covered three domains: family support, friend support, and other support. A Likert 7-point scale was used, ranging from “strong disagreement” to “strong agreement”, and the total score ranged from 12 to 84 points. The higher the score, the better the social support the individual receives. The Cronbach’s α coefficient of the scale in this study was 0.927.

#### Social well-being

The Memorial University of Newfoundland Scale of Happiness (MUNSH) was used to measure the social well-being of older adults in this study [31]. It consists of 24 items. Each item was scored on a three-point Likert scale, with a maximum achievable score of 48. The higher the total score, the better the individual’s well-being. The Cronbach’s α coefficient was 0.879.

#### Internet use

Venkatesh, in developing the Technology Acceptance Model 3, suggested using the question “On average, how much time do you spend on the system each day?” to measure older adults’ Internet use [26]. In this study, we followed this questioning approach to measure older adults’ Internet use.

#### Cognitive impairment

The Short Portable Mental Status Questionnaire (SPMSQ) was used to assess cognitive function in older adults [32]. It consists of 10 items and 3 domains, including orientation, memory, and concentration. The total score ranged from 0 to 10 points. 0–2 errors represent normal cognitive functioning; 3–10 errors represent varying degrees of cognitive impairment.

### Data collection

Data collection was conducted from February 2023 to January 2024. After obtaining approval from the nursing home, the investigation team entered the site for data collection. All investigators received comprehensive training in data collection techniques and were equipped with a standardized set of protocols for dialog with potential participants. During the process of implementation, the investigators firstly explained the purpose of this study and got the written consent from the nursing home residents. For subjective data, such as social isolation, social network, social support, and social well-being. If participants had difficulty completing the questionnaires, the investigators read each questionnaire item to the participants and recorded responses verbatim. For objective data, such as the Internet usage time. With the consent of the participants, the “screen time” option in their smartphone settings was checked and recorded.

### Data analysis

IBM SPSS 26.0 and AMOS Statistics 23.0 were used for statistical analysis. Descriptive statistics were used to analyze participants’ demographics. Numerical variables were presented as means (*M*) and standard deviations (*SD*), and categorical variables as percentages. Skewness and kurtosis were used to test the normality of numerical variables. The scores of social isolation of the older adults with different characteristics were compared by an independent sample *t*-test and ANOVA. Pearson correlation analysis was used to analyze the correlation between social isolation, social network, social support, social well-being, and Internet use. The variables with statistical significance in the single-factor analysis and Internet use were used as independent variables, while social network, social support, and social well-being were used as mediating variables. A multiple-stepwise linear regression analysis was then carried out, with the score of social isolation as the dependent variable. The chain mediating effect of social network, social support, and social well-being on Internet use and social isolation was tested by path analysis. The bootstrap was used 5000 times within the 95% confidence interval to test the significance of the direct, indirect, and total effects of the modified model. Acceptable model fit was determined by consideration of fit indices (i.e., CFI > 0.9, GFI > 0.9, RMSEA < 0.08, $\chi^{\mathit2}$/*df* < 3).

### Ethical consideration

Ethical approval was obtained from the Biological and Medical Research Ethics Committee of the corresponding author’s institution before the study commenced. All methods were performed in accordance with the Declaration of Helsinki. Before signing the written informed consent form, the participants fully understood the purpose, process, and benefits of the study, as well as their right to withdraw from the study at any time.

## Results

### Sample characteristics

Participants’ characteristics were reported in Table 1. The mean age of the participants was 84.75 ± 6.70 years. Most were female (*n* = 288, 65.3%). The majority of them were urban residents (*n* = 376, 85.3%), had multiple children (*n* = 358, 81.2%), were widowed (*n* = 281, 63.7%), and had retired from a public institution (*n* = 203, 46.0%). More than half of the participants (*n* = 256, 58.0%) completed a high school education or higher. 35.6% of participants spent more than $691 per month. Only 7.7% had low socio-economic status. More than half of the participants rated themselves as being in general health (*n* = 236, 53.5%) and 78.7% had any chronic diseases. 59.9% participants were mentally prepared before admission to nursing homes and 51.2% lived with others. More than half used the Internet, and 55.6% had smartphones.

### Questionnaire scores

For numerical variables, the mean of social isolation was 13.47 (SD = 3.58); the average social network score was 14.32 (SD = 4.72); the mean of social support was 51.62 (SD = 11.01); the average social well-being score was 29.44 (SD = 9.38). Mean daily hours of Internet use for nursing home residents was 1.17 (SD = 1.70). The absolute value of skewness of all variables was < 3, and the absolute value of kurtosis was < 8, which was consistent with the normality of the variables [33] (Table 1).

### Single-factor analysis of social isolation among nursing home residents

The social isolation score differences in the nursing home residents of different registered residence, education levels, marital status, number of children, type of pre-retirement occupation, socio-economic status, self-rated health status, admission preparation, residential form, technical devices and Internet access were statistically significant (*P* <.05) (Table 1).

**Table 1:** Distribution of sample characteristics (single-factor analysis) (N=441

Variables	Total (*N* = 441)	Social isolation		*P*-value
		Score	t/F	
Demographic characteristics				
Gender				
Male	153(34.7%)	13.15 ± 3.44	1.86	0.173
Female	288(65.3%)	13.64 ± 3.65		
Registered residence				
Rural	65(14.7%)	12.20 ± 3.74	9.76	0.002
Urban	376(85.3%)	13.69 ± 3.51		
Education level				
Primary school or below	93(21.1%)	12.19 ± 3.67	3.92	0.004
Junior middle school	92(20.9%)	13.95 ± 3.08		
Senior high school	161(36.5%)	13.78 ± 3.39		
Tertiary or above	95(21.5%)	13.72 ± 4.03		
Marital status				
Unmarried	8(1.8%)	9.25 ± 0.89	5.53	< 0.001
Married	138(31.3%)	14.00 ± 3.40		
Divorce	14(3.2%)	14.50 ± 3.82		
Widowed	281(63.7%)	13.28 ± 3.62		
Number of existing children				
0	17(3.9%)	11.76 ± 4.78	3.88	0.009
1	66(15.0%)	13.20 ± 3.40		
2	212(48.1%)	13.19 ± 3.59		
≥3	146(33.1%)	14.19 ± 3.59		
Pre-retirement occupation			9.29	< 0.001
Government department staff	35(7.9%)	12.43 ± 3.89		
Public institution staff	203(46.0%)	14.03 ± 3.61		
Enterprise staff	144(32.7%)	13.31 ± 3.14		
Individual operation	26(5.9%)	15.00 ± 3.84		
Other	33(7.5%)	10.58 ± 2.92		
Economic characteristics				
Monthly fee (US $)			1.37	0.24
< 414	30(6.8%)	12.93 ± 2.67		
414 ~ 552	105(23.8%)	13.40 ± 3.31		
553 ~ 690	149(33.8%)	13.68 ± 3.35		
≥691	157(35.6%)	13.55 ± 4.05		
Socio-economic status			5.83	0.003
Upper	200(45.4%)	13.85 ± 3.72		
Middle	207(46.9%)	13.41 ± 3.53		
Lower	34(7.7%)	11.62 ± 2.31		
Health characteristics				
Any chronic diseases			0.04	0.85
Yes	347(78.7%)	13.45 ± 3.57		
No	94(21.3%)	13.53 ± 3.65		
Self-rated health status			14.55	< 0.001
Very poor	9(2.0%)	12.00 ± 2.24		
Poor	68(15.4%)	11.82 ± 3.22		
General	236(53.5%)	13.03 ± 3.47		
Good	105(23.8%)	15.15 ± 3.49		
Excellent	23(5.2%)	15.74 ± 2.65		
Residential characteristics				
Admission preparation			59.41	< 0.001
Yes	264(59.9%)	14.48 ± 3.34		
No	177(40.1%)	11.96 ± 3.4		
Residential form			9.36	< 0.001
Live with family members	101(22.9%)	14.5 ± 3.31		
Live with others	226(51.2%)	12.79 ± 3.53		
Live alone	114(25.9%)	13.9 ± 3.66		
Technology characteristics				
Technical devices			24.99	< 0.001
Smartphone	245(55.6%)	14.41 ± 3.61		
Senior cell phone	165(37.4%)	12.58 ± 3.07		
No cell phone	31(7.0%)	10.74 ± 3.41		
Internet access			17.65	< 0.001
Using Internet	246(55.8%)	14.48 ± 3.58		
With Internet access but no Internet use	58 (13.2%)	12.60 ± 2.97		
No Internet access	127 (28.8%)	12.17 ± 3.25		
Unclear	10 (2.2%)	10.30 ± 2.83		

Numerical variables	***M*** ± ***SD***	Median(Max, Min)	Skewness	Kurtosis
Age	84.75 ± 6.70	86 (96, 60)	0.335	−0.172
Internet use	1.17 ± 1.70	0.5 (8, 0)	1.791	2.748
Social isolation	13.47 ± 3.58	14 (23, 2)	−0.133	−0.106
Social network	14.32 ± 4.72	14 (27, 3)	0.192	−0.247
Social support	51.62 ± 11.01	51 (79, 18)	0.119	−0.441
Social well-being	29.44 ± 9.38	30 (46, 6)	−0.444	−0.465
Perceived technical usefulness	15.14 ± 7.70	16 (4, 28)	0.016	−1.384
Perceived ease of use	11.38 ± 6.91	10 (4, 28)	0.493	−1.029

### Correlations analysis of the study variables

Table 2 shows correlations for the key study variables. Pearson’s bivariate correlation analyses demonstrated that the dependent variable (social isolation) was significantly positively associated (*r* =.317) with the independent variable (Internet use). All mediating variables (social network, social support, social well-being) were significantly (*p* <.01) and positively associated with the dependent and independent variables.

**Table 2:** Correlations between the study variables (*N* = 441)

	1	2	3	4	5
1. Internet use	1				
2. Social isolation	0.317**	1			
3. Social network	0.447**	0.722**	1		
4. Social support	0.377**	0.631**	0.610**	1	
5. Social well-being	0.293**	0.691**	0.605**	0.576**	1

**p <.01

### Multiple Stepwise linear regression modeling

Before performing regression analysis, we checked the multicollinearity among variables. The variance inflation factors ranged from 1.14 to 3.32. The Durbin–Watson value was 2.17, which indicated no autocorrelation between the residuals. Pearson correlation coefficients of the key study variables were below 0.8. Thus, there was no multicollinearity in the data.

The results from the multiple stepwise linear regression analysis are presented in Table 3. The statistically significant participant characteristics from the single-factor analysis were entered in Model 1. However, these only explained 35.6% of social isolation (*F* = 8.123, *p* <.001). The control variable (marital status, children, pre-retirement occupation, health, and admission preparation) significantly affected social isolation. When Internet use (independent variable) was added into the equation, the variance explained by Model 2 was 37.1% of social isolation (*F* = 9.944, *p* <.01). Model 3 added the mediating variables (social network, social support and social well-being) to the regression equation. The results showed that the mediating variables had significant positive effects on social isolation score after excluding the interference of control variables.

**Table 3:** Factors influencing social isolation

	Model 1	Model 2	Model 3
	B	B	B
Control Variable			
Registered residence (0 = Rural)			
Urban	0.299	0.246	−0.512
Education level (0 = Primary school or below)			
Junior middle school	0.423	0.401	0.573
Senior high school	0.297	0.241	0.389
Tertiary or above	0.973	0.871	0.934
Marital status (0 = Widowed)			
Unmarried	−4.182*	−3.627*	−0.67
Married	0.297	0.244	0.767*
Divorce	1.154	0.927	0.07
Number of existing children (0 = ≥ 3)			
0	−1.478	−2.395**	−0.92
1	−1.12*	−1.399**	−0.356
2	−1.123***	−1.207***	−0.361
Pre-retirement occupation (0 = Public institution staff)			
Government department staff	−1.392*	−1.192*	−0.414
Enterprise staff	−0.617	−0.747	−0.381
Individual operation	0.955	0.874	−0.295
Other	−2.091***	−2.178*	−1.325**
Socio-economic status (0 = Lower)			
Upper	−0.499	−0.579	−0.528
Middle	0.216	0.293	0.099
Self-rated health status (0 = Excellent)			
Very poor	−0.879	−0.449	0.843
Poor	−2.041*	−1.612*	−0.354
General	−1.042	−0.684	0.06
Good	0.927	1.297	1.702**
Admission preparation (0 = No)			
Yes	1.238***	1.226***	0.281
Residential form (0 = Live with family members)			
Live with others	−0.436	−0.476	0.754
Live alone	−0.145	−0.252	1.074
Technical devices (0 = Smartphone)			
Senior cell phone	0.213	0.326	0.321
No cell phone	−0.79	−0.647	−0.252
Internet access (0 = No Internet access)			
Using Internet	1.163	1.031	0.972
With Internet access but no Internet use	0.239	0.302	0.295
Unclear	−1.387	−1.037	−1.013
Independent variable			
Internet use		0.375**	0.159
Mediating variables			
Social network			0.559***
Social support			0.161**
Social well-being			0.223***
R^2^	0.356	0.371	0.522
ΔR^2^	0.356	0.015	0.151
*F*	8.123***	9.944**	78.192***

*p <.05, **p <.01, ***p <.001

### Mediation analysis

**Fig. 2:**
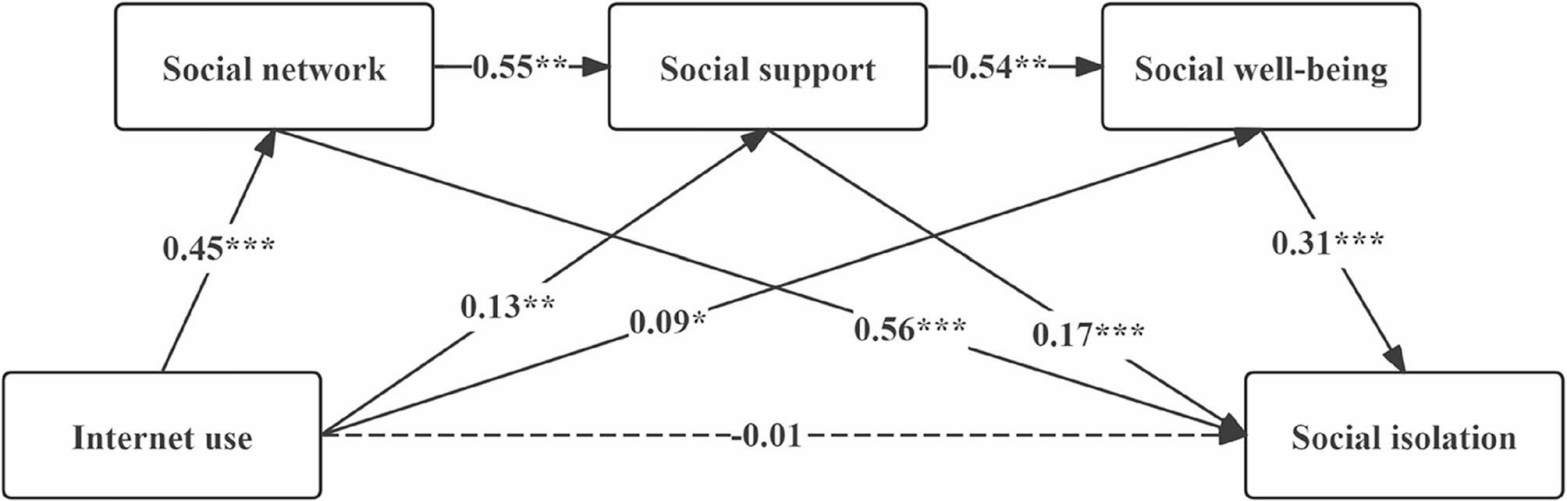
Standardized coefficients of the chain mediating effect model. * *p* <.05, ** *p* <.01, ***p <.001

Our hypothesized mediation model showed acceptable fit indexes (CFI = 0.949, GFI = 0.941, RMSEA = 0.045, $\chi^{\mathit2}$/*df* = 2.329). As shown in Table 4, there were 8 pathways that had statistical significance (*p* <.05). Under standardized regression coefficients, social network (*β* = 0.559, *P* <.001), social support (*β* = 0.165, *P* <.001), and social well-being (*β* = 0.307, *P* <.001) can all have a significant positive effect on social isolation. Internet use has a non-significant effect on social isolation (*β* = −0.015, *P* =.718), but a significant positive effect on social network (*β* = 0.447, *P* <.001), social support (*β* = 0.131, *P* =.002), and social well-being (*β* = 0.088, *P* =.035). Figure 2 demonstrates the fully mediated model with standardized coefficients. Table 5 presented the total, direct and indirect effects of Internet use on social isolation and the associated 95% bootstrap confidence interval. Internet use was found to have a significant total (effect size = 0.392, 95%CI = 0.137 ~ 0.648) and indirect effect (effect size = 0.407, 95%CI = 0.274 ~ 0.541). The indirect effect of Internet use on social isolation was significantly exerted through social network (effect size = 0.252, 95%CI = 0.131 ~ 0.373), social support (effect size = 0.022, 95%CI = 0.011 ~ 0.033), social well-being (effect size = 0.028, 95%CI = 0.013 ~ 0.043), and the chain mediating pathways among them (effect size = 0.041, 95%CI = 0.028 ~ 0.054).

**Table 4:** Path analysis of factors affecting social isolation

Path relationship			Non-standardized coefficient				Standardized coefficients
			Estimate	S.E.	C.*R*.	*P*	Estimate
Social network	<---	Internet use	1.239	0.118	10.468	< 0.001	0.447
Social support	<---	Internet use	0.85	0.271	3.138	0.002	0.131
Social support	<---	Social network	1.287	0.098	13.201	< 0.001	0.551
Social well-being	<---	Internet use	0.488	0.231	2.111	0.035	0.088
Social well-being	<---	Social support	0.463	0.036	12.977	< 0.001	0.543
Social isolation	<---	Internet use	−0.081	0.224	−0.361	0.718	−0.015
Social isolation	<---	Social network	0.409	0.026	15.67	< 0.001	0.559
Social isolation	<---	Social support	0.052	0.012	4.242	< 0.001	0.165
Social isolation	<---	Social well-being	0.113	0.012	9.232	< 0.001	0.307

**Table 5:** Total, direct, and indirect effects of internet use on social isolation

Parameter	Estimate	Bootstrap 95% CI		*P*	% of total effect
		Lower	Upper		
Total effect	0.392	0.137	0.648	0.003	-
Direct effect	−0.015	−0.155	0.124	0.798	−3.83%
Total indirect effect	0.407	0.274	0.541	< 0.001	103.83%
Internet use →Social network → Social isolation	0.252	0.131	0.373	< 0.001	64.28%
Internet use → Social support → Social isolation	0.022	0.011	0.033	0.032	5.61%
Internet use → Social well-being → Social isolation	0.028	0.013	0.043	0.028	7.14%
Internet use → Social network → Social support → Social isolation	0.042	0.031	0.053	0.013	10.71%
Internet use →Social support → Social well-being → Social isolation	0.022	0.009	0.035	0.034	5.61%
Internet use → Social network → Social support → Social well-being → Social isolation	0.041	0.028	0.054	0.017	10.46%

## Discussion

Guided by the convoy model, this study explored the factors of social isolation and tested social network, social support, and social well-being as mediators of the relationship between Internet use and social isolation among nursing home residents, which was rarely studied before. This study reflected, to some extent, the current status of social isolation, Internet use, and social relationships among nursing home residents in China. Our results highlighted the necessity of helping older adults alleviate social isolation through increased Internet use. Findings from this study supported the extensibility of the convoy model as it evolves in the digital era, that was clarified the impact of Internet use on older adults’ social isolation.

In recent years, social isolation has received extensive attention in senior care, as it has a clear impact on the health of older adults. Our results showed that the social isolation score of nursing home residents was 13.47 ± 3.58, which was lower than the social isolation score of 19.00 ± 3.80 and 14.15 ± 3.73 for older adults in the Chinese community reported by Pang [28] and Wang et al. [34]. This result was not optimistic, suggesting that nursing home residents may be at a higher risk of experiencing social isolation. This phenomenon may be the result of the combined effect of multiple factors. First, after entering a nursing home, the older adult’s existing social relationships tend to fade rapidly due to a sharp decline in contact frequency [35]. Second, the physical condition of nursing home residents is not optimistic. Chronic illnesses, advanced age, disability, and dementia are objective factors that directly limit their social participation [36]. Third, some older adults who are placed in nursing homes against their will may develop a sense of abandonment, leading to diminished self-worth. This negative cognition can also cause them to choose self-isolation, making them reluctant to engage in deep interactions with neighbors [37]. The mean hours of Internet use for nursing home residents in this study were 1.17 ± 1.70, which is similar to the data reported by Hu et al. [38]. 58.1% of the participants had Internet access experience, which is basically in line with the latest national data (57.5%) published by the China Internet Network Information Center (CNNIC) [39]. Despite the fact that the rate of digital device ownership among older adults in China has been increasing year by year. However, they are still not active enough to surf the Internet. The results of this study highlighted that Internet use would have a positive impact on the social relationships of nursing home residents. Therefore, future research focusing on improving digital literacy and Internet usage among older adults is needed to alleviate social isolation.

In multiple stepwise linear regression analysis, we examined the multiple influence factors on social isolation. The participants without spouses were more likely to experience social isolation than married. This may be explained that married older adults appear to be more psychologically resilient in coping with the risk of social isolation. Mapoma et al. demonstrated that mutual support between spouses tends to make them more comfortable in the face of such life-environmental migrations [40]. This study pointed out that the number of children can affect social isolation. Influenced by the traditional Chinese cultural concept of “more children, more well-being”, more than 4/5 of the participants in this study had two or more children. Therefore, Chinese older adults may score higher on the objective connectedness dimension of the SIS because of a larger number of family members. This also seemed to explain the higher SIS scores of older adults in China compared to abroad. Park et al.’s results explained that the number of children can affect the level of intergenerational family contact, and those older adults with poorer family contact are more likely to experience social isolation [41]. Health status was also an important factor influencing the social isolation of nursing home residents. The participants with poorer self-rated health had greater social isolation. This may be because physical dysfunction, chronic diseases, and mental health issues can all limit older adults’ social activities and increase the risk of social isolation. Kristensen et al. research also confirmed that poor physical health status not only restricted older adults’ physical activity, but also narrowed their social networks and further affected the quality of social relationships [42]. This study found that older adults who were psychologically prepared prior to admission had a lower risk of social isolation, which was an influencing factor not commonly found in previous studies. Davies et al. attributed this phenomenon to an individual’s emotional identification and acceptance of being admitted to a nursing home [43]. Active residents had a high level of acceptance and identification with the lifestyle and environment of the nursing home. They were able to adapt positively to the pace of life, took the initiative to participate in various activities organized by the nursing homes, and established new social relationships with other older adults, thus reducing social isolation. In combination with the conclusion of this study that Internet use exerts a positive impact on social isolation, we can foresee that providing nursing home integration support interventions for residents through digital media will be imperative in the future.

In general, on the foundation of the convoy model and results of previous multivariate analysis, our finding revealed the influencing factors of social isolation, as well as the relationship between social isolation and Internet use. After controlling for confounding factors interference, we found that Internet use can positively affect social isolation scores. This was consistent with the results by Silva et al. [44], who investigated information on older adults from 17 countries and found that Internet use would mitigate social isolation after controlling for socio-demographic, economic, and health characteristics of older adults. However, the direct effect of Internet use on social isolation was not significant in the path analysis. Instead, it was fully mediated by social network, social support, and social well-being. This result suggested that we should focus on the development of online social skills when promoting Internet use among nursing home residents. Increasing the hours of Internet use alone did not have a significant mitigating effect on social isolation. Meshi et al. came to a similar conclusion and suggested that hours of Internet use were not a reliable predictor of social isolation among older adults [13]. Meanwhile, they also pointed to the proper use of online social media as key to influencing social isolation.

Our results suggested that social networks had the greatest mediating effect between Internet use and social isolation. This was similar to the findings of Wang et al. [45], who confirmed the key role played by social networks in the relationship between Internet use and mental health. A reasonable explanation is that a strong social network would not only increase the level of objective social connections of older adults, but also provide them with emotional support and psychological comfort. This overall enhancement, both at the subjective and objective levels, was more beneficial for older adults in maintaining a positive mental attitude and reducing the negative emotions associated with social isolation [46].

Our study further indicated that social support plays a significant mediating role in the relationship between Internet use and reduced social isolation. This finding aligns with the work of Kusumota et al. [47], who reported that Internet use facilitates social support, enhancing older adults’ sense of belonging and alleviating their loneliness. Building on our regression analysis, which identified the number of children as a key factor influencing social isolation, we propose that intergenerational digital support represents a promising new intervention paradigm for mitigating isolation in nursing homes. This concept is supported by the work of Cronan et al. [48], who successfully alleviated social isolation by establishing online connections between nursing home residents and middle school students through intergenerational video conferencing. Therefore, future research should explore not only leveraging digital media to create novel intergenerational social channels for residents but also investigating how such reciprocal digital exchanges can simultaneously enhance older adults’ digital literacy through peer learning and other ongoing support.

Additionally, social well-being also served as a mediating variable in the relationship between Internet use and social isolation. This was consistent with Xu et al. [49]. Social isolation among older adults is not merely a “quantitative deficiency in social interactions” but, more fundamentally, a “deficit in emotional bonding”. Critically, this study identified a chain mediation pathway: Internet use alleviates social isolation by first strengthening social connections and subsequently enhancing social well-being. A reasonable explanation is that Internet use (such as video calls and sharing daily life online) directly expands older adults’ social networks. This can meet older adults’ emotional needs (such as feeling cared for and experiencing a sense of belonging), thereby enhancing their social well-being. Individuals with high levels of social well-being are more likely to proactively initiate or respond to social interactions, thereby breaking the cycle of social isolation [50]. This insight holds particular significance for nursing home residents facing mobility constraints and limited social circles.

This study has important implications for promoting digital inclusion and alleviating social isolation among nursing home residents. The findings provide healthcare providers, policymakers, and senior care administrators with a comprehensive understanding of how Internet use influences social connectedness in this vulnerable population. Crucially, it suggests that facilitating Internet use represents a practical, scalable, and cost-effective strategy to combat isolation among older adults. To translate these insights into tangible benefits, targeted initiatives are essential. Prioritizing programs that strengthen digital literacy and social engagement skills through intergenerational support could enhance adoption and sustained use. Furthermore, this research provides empirical validation for the positive role of Internet use in reducing perceived isolation, reinforcing the need to integrate digital inclusion into broader strategies for healthy aging and quality of life in residential care settings.

## Limitations

This study has several limitations. First, its cross-sectional design is susceptible to confounding bias and prevents confirmation of causal relationships between the assessed factors. Second, the reliance on convenience sampling limits generalizability. Data were collected from nursing home residents in more developed regions of eastern coastal China; consequently, the findings may not reflect the potentially greater challenges related to Internet access and social isolation faced by residents in underdeveloped regions. Future nationwide studies employing multi-center stratified random sampling and longitudinal designs are necessary to validate these conclusions. Finally, the use of self-reported data introduces potential information bias. Incorporating more objective outcome indicators in future research could help mitigate this limitation.

## Conclusions

Based on the convoy model, the structural equation model of Internet use affecting social isolation was developed. Social networks, social support, and social well-being were important mediating factors. In addition, differences in marital status, number of children, pre-retirement occupation, health status, and psychological preparation for admission also have an impact on social isolation. Therefore, nursing home healthcare staff should focus on these influences in order to effectively alleviate social isolation among nursing home residents.

## Supplementary Information

Supplementary Material 1.

## Data Availability

The datasets used and/or analyzed during the current study are available from the corresponding author on reasonable request.
